# De novo assembly and annotation of the retinal transcriptome for the Nile grass rat (*Arvicanthis ansorgei*)

**DOI:** 10.1371/journal.pone.0179061

**Published:** 2017-07-31

**Authors:** Melissa M. Liu, Michael Farkas, Perrine Spinnhirny, Paul Pevet, Eric Pierce, David Hicks, Donald J. Zack

**Affiliations:** 1 Wilmer Eye Institute, Johns Hopkins University School of Medicine, Baltimore, MD, United States of America; 2 Department of Ophthalmology, Jacobs School of Medicine and Biomedical Sciences, University at Buffalo, Buffalo, NY, United States of America; 3 Research Service, Veterans Administration Western New York Healthcare System, Buffalo, NY, United States of America; 4 Institut des Neurosciences Cellulaires et Intégratives, CNRS UPR3212, Strasbourg, France; 5 Ocular Genomics Institute, Department of Ophthalmology, Massachusetts Eye and Ear Infirmary, Boston, MA, United States of America; 6 Department of Molecular Biology and Genetics, Johns Hopkins University School of Medicine, Baltimore, MD, United States of America; 7 Department of Neuroscience, Johns Hopkins University School of Medicine, Baltimore, MD, United States of America; 8 McKusick-Nathans Institute of Genetic Medicine, Johns Hopkins University School of Medicine, Baltimore, MD, United States of America; 9 Institut de la Vision, Université Pierre et Marie Curie, Paris, France; University of Florida, UNITED STATES

## Abstract

Cone photoreceptors are required for color vision and high acuity vision, and they die in a variety of retinal degenerations, leading to irreversible vision loss and reduced quality of life. To date, there are no approved therapies that promote the health and survival of cones. The development of novel treatments targeting cones has been challenging and impeded, in part, by the limitations inherent in using common rodent model organisms, which are nocturnal and rod-dominant, to study cone biology. The African Nile grass rat (*Arvicanthis ansorgei*), a diurnal animal whose photoreceptor population is more than 30% cones, offers significant potential as a model organism for the study of cone development, biology, and degeneration. However, a significant limitation in using the *A*. *ansorgei* retina for molecular studies is that *A*. *ansorgei* does not have a sequenced genome or transcriptome. Here we present the first de novo assembled and functionally annotated transcriptome for *A*. *ansorgei*. We performed RNA sequencing for *A*. *ansorgei* whole retina to a depth of 321 million pairs of reads and assembled 400,584 Trinity transcripts. Transcriptome-wide analyses and annotations suggest that our data set confers nearly full length coverage for the majority of retinal transcripts. Our high quality annotated transcriptome is publicly available, and we hope it will facilitate wider usage of *A*. *ansorgei* as a model organism for molecular studies of cone biology and retinal degeneration.

## Introduction

Rod and cone photoreceptors are the light sensitive cells of the retina that enable the detection of visual stimuli. Rods are responsible for vision under dim light conditions, whereas cones mediate color and high acuity vision. Cone photoreceptors degenerate in a variety of eye diseases, including age-related macular degeneration (AMD), cone-rod dystrophy, and retinitis pigmentosa (RP). Development of therapeutic strategies promoting the survival of cones in these pathological settings has been challenging, due in part to difficulties inherent in studying cones when using common rodent model organisms. Laboratory mice (*Mus musculus*) and rats (*Rattus norvegicus*) are nocturnal and have rod-dominant retinas, with cones comprising only ~3% and ~1% of *M*. *musculus*[1] and *R*. *norvegicus*[2] photoreceptors, respectively. Thus, these organisms are not ideally suited for studies of cones. With the goal of developing improved small animal models for the study of cones, efforts have been made to identify rodents that contain more cone-enriched retinas. Among the rodent species that have been identified as having cone-enriched retinas are the African Nile grass rat (*Arvicanthis ansorgei*) [3] and the 13-lined ground squirrel (*Ictidomys tridecemlineatus*) [4].

From the experimental perspective, *A*. *ansorgei* has the advantage over *I*. *tridecemlineatus* in that it can be more easily maintained in laboratory colonies. Until recently, *I*. *tridecemlineatus* could not be bred under laboratory conditions and thus had to be caught wild. Although a protocol has since been established for maintaining *I*. *tridecemlineatus* in laboratory colonies, there are unique challenges related to caring for animals that undergo months of torpor [5]. *A*. *ansorgei* has primarily been studied in the context of circadian rhythms [3]. As its retina is comprised of more than 30% cones, it is suitable as a mammalian model for the study of cone biology and pathology [6]. As one example, the N-methyl-N-nitrosourea (MNU) chemical induced retinal degeneration model has been established in *A*. *ansorgei*. Structural and functional studies demonstrate that MNU treatment causes a spatiotemporally reproducible photoreceptor degeneration in the *A*. *ansorgei* retina [7]. The pattern of degeneration is one in which rod cell death is followed by cone cell death, a pattern of degeneration that is also seen in human RP. Thus, *A*. *ansorgei* is well-suited as a model for molecular studies of cone function and degeneration and for the identification of cone specific genes, pathways, and mechanisms that promote homeostasis and survival.

A significant hurdle for molecular studies in *A*. *ansorgei*, however, is that there is very limited genome or transcriptome data currently available for this organism. Due to insufficient species specific sequence information, the research community has had to characterize genes of interest one at a time or rely on data from *M*. *musculus* or *R*. *rattus*, which generally does not completely or accurately represent *A*. *ansorgei*. Especially in the context of cone photoreceptor studies, there are likely mechanisms in the diurnal *A*. *ansorgei* that would be missed when basing such studies on information gleaned entirely from nocturnal rod-dominant model organisms.

As of February 2017, there were less than 400 ESTs in the NCBI repository for the entire *Arvicanthis* genus, whereas there are 4.9 million ESTs for *M*. *musculus* and 1.1 million ESTs for *R*. *norvegicus*. The genus Arvicanthis, which has seven recognized species [8], has no genomic sequencing data, and only one RNA sequencing data set has been published, which was for the species *A*. *niloticus* [9]. Phylogenetic analysis based on both mitochondrial and nuclear genes has revealed that within the genus, there are two main clades, where *A*. *niloticus* is a member of one, and *A*. *ansorgei* is a member of the other [10]. The evolutionary event dividing the genus into these two sister monophyletic subgroups is estimated to have occurred more than 5 million years ago [11]. With respect to diversity at the level of the DNA sequence, analysis of the complete sequence of the highly conserved gene encoding cytochrome b has demonstrated that the average degree of sequence divergence between different species of the Arvicanthis genus is 15.5% [11].

To aid in the further development of *A*. *ansorgei* as a useful model for studies of cone development, function, and degeneration, we performed RNA-Seq on retinas from adult animals and de novo assembled and annotated the first transcriptome for this species. The assembled and annotated retinal transcriptome is publicly available and will hopefully serve as a resource for downstream molecular studies.

## Methods

### RNA preparation

All animals were maintained in compliance with the guidelines of the Animal Care and Use Committee of Institut des Neurosciences Cellulaires et Intégratives (Chronobiotron UMR 3415). The protocols used in this study were approved by the Comité Régional d'Ethique en Matière d'Expérimentation Animale de Strasbourg (CREMEAS, ethical license reference AL/24/31/02/13). *A*. *ansorgei* were housed in 22±2°C rooms under a 12:12 hour light dark cycle, 100 lux white light with lights on at 7 am and lights off at 7 pm. Animals were fed with standard rat chow supplied *ad libitum*. Young adult (5–6 months) female Arvicanthis (n = 2) were used for this study. Euthanasia was performed by CO_2_ inhalation, and all efforts were taken to minimize suffering. Whole retinas were rapidly isolated by cutting across the cornea with a clean scalpel blade followed by retinal extrusion. They were immediately flash frozen in liquid nitrogen and stored at -80°C until ready for use. Retinas were independently homogenized in Buffer RLT Plus + 1% β-mercaptoethanol, and total RNA was extracted with genomic DNA removal using the RNeasy Plus Mini Kit according to manufacturer’s instructions (Qiagen, Germantown, MD, USA). RNA samples were quantified by the RNA 6000 Nano Kit on the 2100 Bioanalyzer (Agilent, Santa Clara, CA, USA).

### RNA-Seq library preparation and sequencing

Two high quality RNA samples were used to prepare independent RNA-Seq libraries using previously described methods [12]. First strand cDNA synthesis was performed with 195 ng total RNA using anchored oligo-dT and SuperScript III First-Strand Synthesis SuperMix (ThermoFisher, Waltham, MA, USA). Second strand cDNA synthesis was peformed using RNase H, DNA Polymerase I, and Invitrogen Second Strand Buffer (ThermoFisher, Waltham, MA, USA). Double stranded cDNA was purified using DNA Clean & Concentrator-5 (Zymo Research, Irvine, CA, USA). Tagmentation was performed using the Nextera DNA Library Preparation Kit (Illumina, San Diego, CA, USA). Tagmented DNA was purified using DNA Clean & Concentrator-5 before Nextera PCR amplification. Libraries were cleaned using Agencourt AMPure XP beads according to manufacturer’s instructions (Beckman Coulter, Brea, CA, USA). Libraries were evaluated by the High Sensitivity DNA Kit on the 2100 Bioanalyzer. The average size of the library fragments were 705 bp and 561 bp for samples S1 and S2, respectively. They were then sequenced with 93 bp paired ends on an Illumina HiSeq 2000 in high output mode with V3 chemistry.

### De novo transcriptome assembly and quantification

FastQC (https://www.bioinformatics.babraham.ac.uk/projects/fastqc/) was used to assess the quality of the sequencing data. Trimmomatic was used to trim adapters and leading or trailing bases with quality score less than 30, and resultant reads less than 25 bp in length were dropped [13]. Trimmomatic was invoked using the command java–jar trimmomatic-0.32.jar PE–threads 10 –phred33 R1.fastq.gz R2.fastq.gz paired_R1.fastq.gz unpaired_R1.fastq.gz paired_R2.fastq.gz unpaired_R2.fastq.gz ILLUMINACLIP:/adapters.fa:2:30:8 LEADING:30 TRAILING:30 MINLEN:25. Cleaned paired reads were concatenated across both samples to form combined left.fastq and right.fastq files. The calculate_stats utility from seq_crumbs was used to calculate Q20 and Q30 using the command calculate_stats–c [*left*.*fastq*,*right*.*fastq*] > out.txt [14]. Cleaned paired reads were combined across both samples and passed to Trinity for de novo transcriptome assembly with in silico normalization [15]. Trinity was invoked using the command Trinity—seqType fq—max_memory 480G —CPU 48—normalize_reads—left left.fastq—right right.fastq—output out_dir—grid_conf trinity_conf.txt. RSEM, with Bowtie alignment, was used to quantify Trinity assembled transcript abundance in each sample [16]. The Trinity utility was invoked using the command align_and_estimate_abundance.pl—transcripts Trinity.fasta—seqType fq—left paired_R1.fastq.gz—right paired_R2.fastq.gz—est_method RSEM—aln_method bowtie—trinity_mode.

### De novo transcriptome functional annotation

TransDecoder was used to search the Trinity assembled transcripts for open reading frames (ORFs) encoding peptides of at least 100 amino acids in length [15]. Trinity transcripts of any length with ORFs homologous to known proteins or containing protein domains were identified by BlastP (v2.2.30) [17] queries against the Swiss-Prot database [18] and HMMER3 [19] queries against the Pfam database [20], respectively. The final TransDecoder-predicted coding regions include those meeting the minimum length criteria and those of any length with BlastP or Pfam homology. Trinotate was then used for functional annotation [15]. The TransDecoder-predicted coding regions were searched for Pfam protein domains using HMMER3, signal peptides using SignalP 4.1 [21], transmembrane regions using TMHMM [22], rRNAs using RNAMMER [23], homology to known proteins using BlastP (v2.2.30) (*E*<10^−5^) against both the Swiss-Prot and the UniRef90 [24] databases, and annotations from gene ontology (GO) [25] and EggNOG [26]. The Trinity transcripts were also searched for homology by BlastX (v2.2.30) (*E*<10^−5^) against the Swiss-Prot and UniRef90 database. All annotations were aggregated in a final report.

### De novo transcriptome evaluation

Trinity transcripts were searched by BlastN and BlastX against the NCBI RefSeq mRNA and protein databases, respectively, for both *M*. *musculus* and *R*. *norvegicus*. The percent coverage along the target transcript or protein was determined using the Trinity provided utility invoked with the command analyze_blastPlus_topHit_coverage.pl blast_result.outfmt6 Trinity.fasta blast_db [15]. For each Blast hit in the target database, the best matching Trinity transcript was selected, and the percent of the Blast hit’s length covered by the Trinity transcript was determined.

Trinity transcripts were filtered for those with BlastX hits against the Swiss-Prot database and ranked by TPM. Gene Set Enrichment Analysis (GSEA) was used to identify Gene Ontology (GO) annotations enriched among these Trinity transcripts with mean TPM>1 [27]. Cytoscape [28] and Enrichment Map [28] were used to visualize the results.

### Phylogenetic analysis

Multiple sequence alignment of the coding sequence (CDS) of representative genes for *A*. *niloticus* and other model organisms was performed with MUSCLE using the UPGMB clustering method [29]. A Neighbor-Joining [30] phylogenetic reconstruction was created using the Maximum Composition Likelihood model [31] to compute evolutionary distances. These analyses were performed in MEGA6 (v6.06) [32].

### Availability of data and materials

The datasets supporting the conclusions of this article are available in the NCBI Sequence Read Archive (SRA) (https://www.ncbi.nlm.nih.gov/sra/), accession numbers SRR5190211 and SRR5190212, and within the article and its Supporting Information files.

## Results

Two RNA samples, S1 and S2, with RNA Integrity Numbers (RINs) of 9.8 and 9.7 (S1 Fig) were independently extracted from the retinas of adult *A*. *ansorgei*. RNA-Seq libraries with broad fragment length distributions peaking at 500–600 bp (S2 Fig) were prepared and sequenced to a depth of 155,722,626 and 165,504,305 pairs of reads. FastQC confirmed adequate per base sequence quality (S3 Fig). After trimming adapters and low-quality bases, 151,032,789 and 161,439,517 cleaned pairs of reads remained. Ultimately, 312,472,306 pairs of reads were passed to Trinity for de novo transcriptome assembly. Trinity assembled 400,584 transcripts and 356,299 unigenes (Table 1). The Trinity transcripts had a mean length of 801 nt and a N50 length of 1,457 nt, meaning 50% of assembled bases are part of Trinity transcripts of length 1,457 nt or greater. There were 69,664 Trinity transcripts with open reading frames of at least 100 amino acids in length, corresponding to 38,908 unigenes, of which 29,716 had a single transcript and 9,192 had multiple transcripts.

**Table 1:** *A*. *ansorgei* transcriptome assembly statistics.

**RNA-Seq reads**	
Pairs of raw reads	321,226,931
Pairs of cleaned reads	312,472,306
Q20	98.3%
Q30	92.1%
**Trinity assembly**	
Total Trinity genes	356,299
Total Trinity transcripts	400,584
Total assembled bases	324,826,766
Percent GC	47.2
N50 length	1,457
Average length	811
Median length	401

Q30: Percent of bases in cleaned reads with quality score 30 or greater; N50: length of longest Trinity transcript such that 50% of bases are in Trinity transcripts of length N50 or greater.

Functional annotation for the Trinity transcripts was performed using the Trinotate annotation pipeline. TransDecoder was used to predict coding regions, which were then searched for Pfam protein domains, signal peptides, transmembrane regions, rRNAs, homology to known proteins in both the Swiss-Prot and the UniRef90 databases, and gene ontology (GO) annotations. The Trinity transcripts were also searched for BlastX homology against the Swiss-Prot and UniRef90 database. Alignment of the RNA-Seq reads to the de novo assembled transcriptome was evaluated. Expression levels of Trinity assembled genes and transcripts were estimated in units of TPM (transcripts per million) using RNA-Seq by Expectation Maximization (RSEM) with Bowtie as the alignment algorithm. The majority of reads aligned to the transcriptome and in proper pairs (Table 2). The sequences, full annotations, and expression levels for the Trinity assembled de novo transcripts are available as Supporting Information files (S1 File and S2 File).

**Table 2:** RNA-Seq read alignment statistics.

	S1	S2
Total reads	151,032,789	161,439,517
Total aligned reads	115,727,548 (76.6%)	115,450,815 (71.5%)
Aligned reads in proper pairs	87,084,546 (75.3%)	81,044,720 (70.2%)
Aligned reads in improper pairs	16,756,080 (14.5%)	25,731,616 (22.3%)
Aligned right read only	6,032,890 (5.2%)	4,391,407 (3.8%)
Aligned left read only	5,854,032 (5.1%)	4,283,072 (3.7%)

Proper pair: left and right reads map to a single Trinity transcript in the correct orientation.

**Fig 1:**
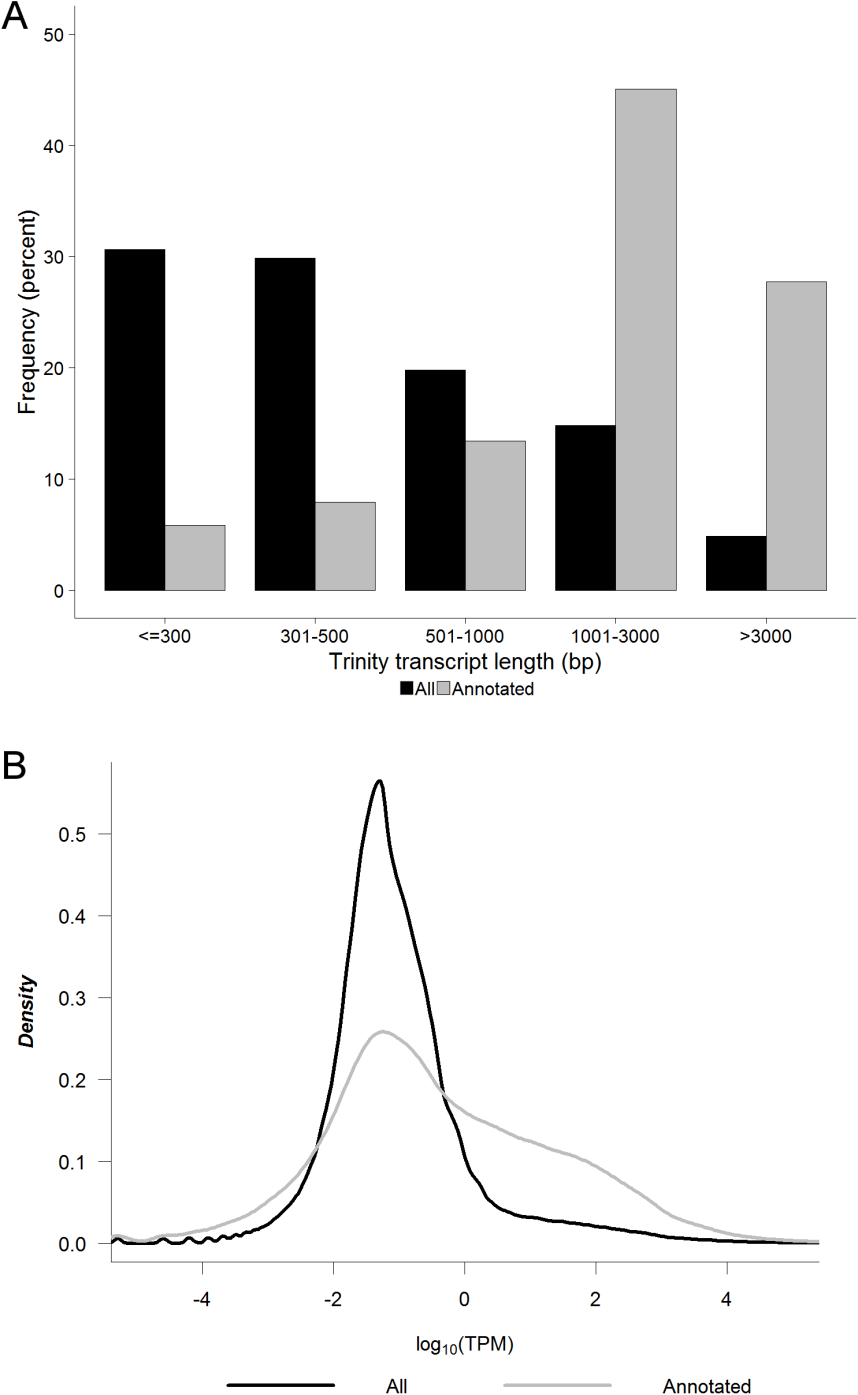
Trinity transcript length and level of expression. A) Length and B) average level of expression between S1 and S2 in units of log10(TPM) (TPM = transcripts per million) for all Trinity transcripts (n = 400,584) and the subset of Trinity transcripts with Swiss-Prot BlastX homology (n = 63,242).

We next examined the length and level of expression of the identified transcripts. Of the 400,584 assembled Trinity transcripts, 78,915 (19.7%) were greater than 1kb in length, and of the subset of 63,242 Trinity transcripts with Swiss-Prot BlastX homology, 46,038 (72.8%) were greater than 1kb in length (Fig 1A). The subset with Swiss-Prot BlastX homology was also more highly expressed, with 35.2% of annotated transcripts being expressed at a level greater than 1 TPM, as compared to only 10.0% of all Trinity transcripts being expressed at this level (Fig 1B). The putative coding transcripts were therefore more likely to be both higher in abundance and longer in length than their non-coding counterparts. Pairwise analysis for the 8,866 orthologous genes expressed at a level greater than 1 TPM (*A*. *ansorgei*) or 1 FPKM (*A*. *niloticus*)[9] showed that global gene expression levels were moderately correlated (Pearson correlation coefficient = 0.63) between these two members of the *Arvicanthis* genus (S4 Fig).

**Fig 2:**
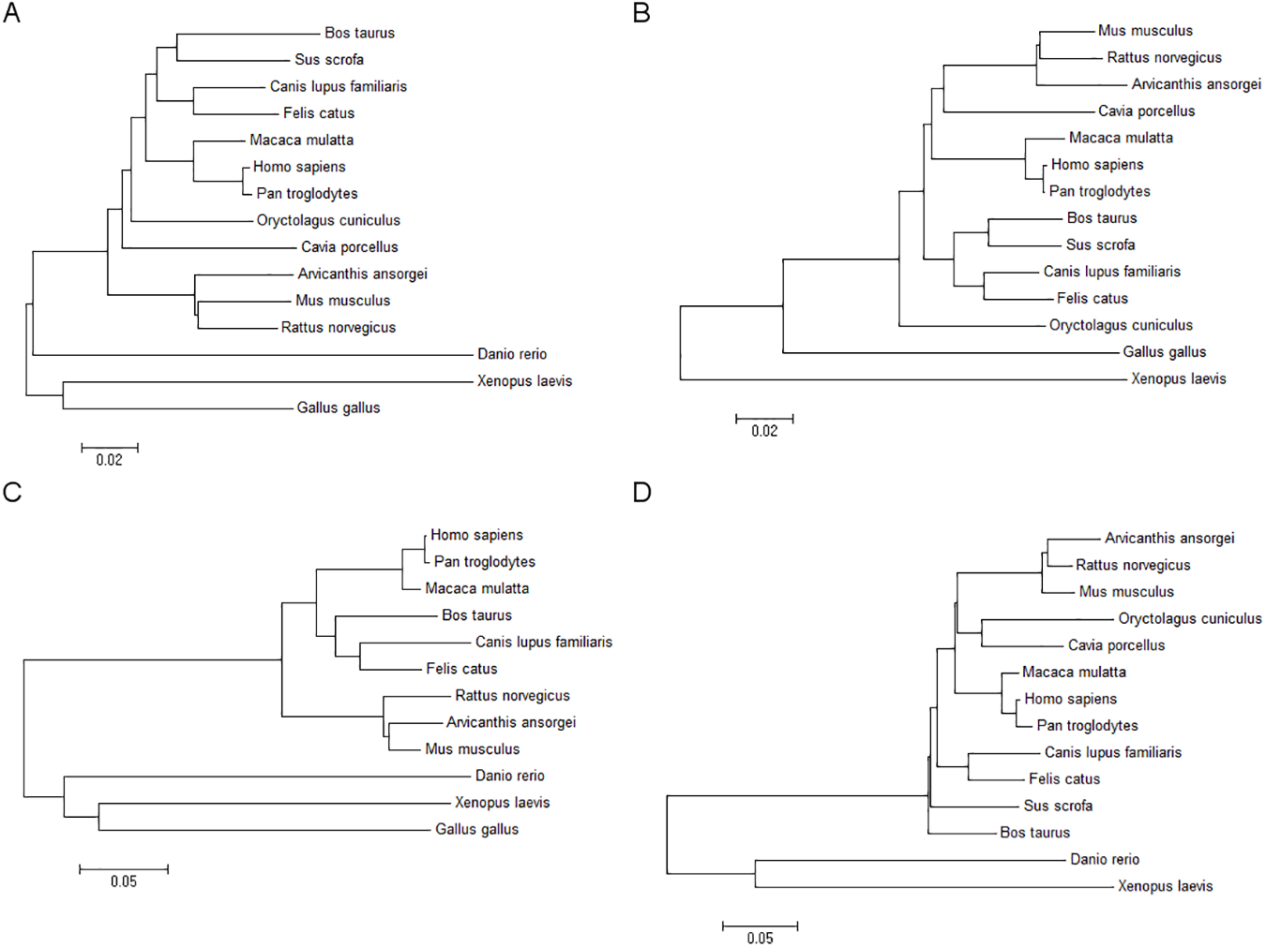
Phylogenetic analysis for retinal genes. Multiple sequence alignment for the CDS of A) rhodopsin B) short-wave-sensitive opsin 1 C) melanopsin D) cone-rod homeobox for *A*. *niloticus* and other model organisms performed using MUSCLE. The Maximum Composition Likelihood model was used to construct Neighbor-Joining phylogenetic trees.

**Fig 3:**
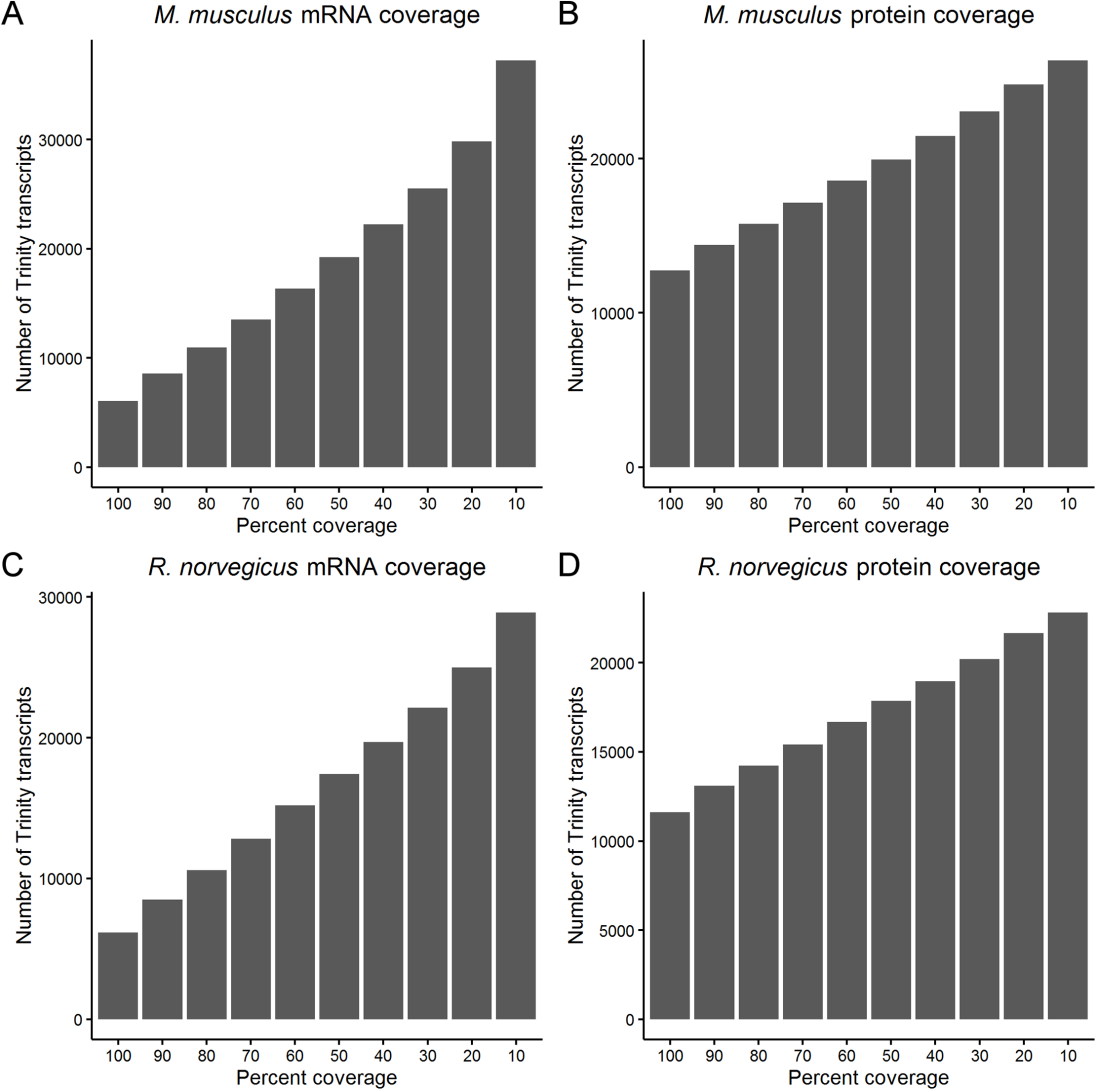
Coverage of *M*. *musculus* and *R*. *norvegicus* mRNA transcripts and proteins. The coverage of *M*. *musculus* A) mRNA and B) protein and *R*. *norvegicus* C) mRNA and D) protein provided by transcripts from the de novo assembled *A*. *ansorgei* transcriptome. For a Trinity transcript in bin of percent coverage *n*, the Trinity transcript covers at least *n*-10% of the length of the target mRNA or protein.

Phylogenetic analysis was performed using the Trinity assembled CDS for four retinal genes in order to place *A*. *ansorgei* in the context of other common model organisms (Fig 2). The topologies of the phylogenetic trees are broadly comparable and place *A*. *ansorgei* in closest proximity to *R*. *norvegicus* and *M*. *musculus*. Consequently, *R*. *norvegicus* and *M*. *musculus* were used as the references for estimating the completeness of the *A*. *ansorgei* de novo assembled transcriptome. To perform this analysis, Blast was used to query each Trinity transcript against the RefSeq databases of mRNAs and proteins for *M*. *musculus* and *R*. *norvegicus*, and for each hit in the target database, the length of the hit covered by the best matching Trinity transcript was determined (Fig 3).

There are 8,551 *M*. *musculus* and 8,486 *R*. *norvegicus* RefSeq mRNAs that have Trinity transcripts which align with at least 80% coverage, and there are 14,397 *M*. *musculus* and 13,095 *R*. *norvegicus* RefSeq proteins that have Trinity transcripts which translate to cover at least 80% of their length. We chose 33 canonical retinal cell specific markers and identified their corresponding Trinity transcripts. The majority show near full length coverage and sequence identity for the coding sequences of the proteins against which they demonstrate BlastX homology (Table 3). Proteins expected to be highly conserved, for example 40S ribosomal proteins, 60S ribosomal proteins, beta actin, and cytochrome c, all demonstrate 100% identity and 100% full length coverage.

**Table 3:** Trinity transcripts with homology for selected retinal markers.

Trinity transcript	BlastX hit	Percent identity	Hit length	Percent hit aligned	Hit description	Average TPM
TR103769|c1_g1_i4	NP_446140.1	95.18	83	100	retinal cone rhodopsin-sensitive cGMP 3',5'-cyclic phosphodiesterase subunit gamma	75.0
TR104093|c0_g2_i3	NP_446278.2	98.35	424	97.7	pyruvate dehydrogenase kinase, isozyme 1 precursor	4.8
TR107698|c10_g1_i2	NP_001100357.1	97.3	185	91.58	guanylyl cyclase-activating protein 1	287.0
TR107698|c8_g2_i1	NP_001101668.1	96.52	201	100	guanylyl cyclase-activating protein 2	256.1
TR113665|c4_g1_i1	NP_446130.1	96.62	207	60.53	retinal homeobox protein Rx	8.5
TR115337|c4_g5_i3	NP_001102250.2	99.14	350	100	guanine nucleotide-binding protein G(t) subunit alpha-1	1483.6
TR116031|c7_g1_i1	NP_446153.1	97.49	1635	82.53	voltage-dependent L-type calcium channel subunit alpha-1F	10.3
TR116046|c3_g2_i3	NP_037133.1	99.76	422	100	paired box protein Pax-6	6.4
TR130418|c8_g2_i3	NP_446283.1	95.67	831	75	retinal guanylyl cyclase 2 precursor	12.5
TR131335|c11_g1_i1	NP_599182.1	100	86	20.87	POU domain, class 4, transcription factor 2	1.3
TR134023|c5_g1_i1	NP_001099506.1	96.64	238	100	neural retina-specific leucine zipper protein	10.5
TR135421|c0_g2_i1	NP_112277.1	96.82	346	100	short-wave-sensitive opsin 1	43.7
TR137704|c6_g9_i2	NP_001101191.1	93.24	518	22.62	retinal-specific ATP-binding cassette transporter	63.9
TR137727|c7_g1_i1	NP_037004.1	94.72	246	100	phosducin	382.4
TR137897|c11_g6_i1	NP_446000.1	96.47	255	71.03	medium-wave-sensitive opsin 1	596.2
TR142441|c12_g1_i1	NP_001099183.1	99.77	442	69.5	protein kinase C alpha type	15.4
TR205210|c0_g1_i1	NP_446240.2	99.65	288	100	syntaxin-1A	2.6
TR55594|c6_g2_i6	NP_112358.1	95.57	564	100	rhodopsin kinase precursor	207.2
TR56231|c0_g1_i2	NP_037069.1	100	202	98.54	beta-crystallin B2	1383.0
TR58523|c4_g1_i2	NP_543177.1	95.79	190	94.06	recoverin	112.1
TR59222|c9_g2_i2	NP_036796.1	99.57	235	76.55	synaptophysin	372.0
TR70411|c9_g2_i6	NP_001101112.1	60.42	141	30.19	tubby-related protein 1	4.8
TR70482|c3_g1_i5	NP_254276.1	94.25	348	100	rhodopsin	4697.7
TR73195|c4_g1_i4	NP_001162599.1	98.89	361	100	visual system homeobox 2	7.9
TR81238|c2_g1_i2	NP_445876.1	99.17	1079	100	electrogenic sodium bicarbonate cotransporter 1	5.8
TR85026|c0_g1_i2	NP_001099744.1	96.21	317	100	retinaldehyde-binding protein 1	187.7
TR87913|c0_g1_i1	NP_620215.1	89.66	474	100	melanopsin	3.5
TR88772|c0_g1_i1	NP_001102651.1	100	319	100	transcription factor SOX-2	7.2
TR92173|c5_g2_i2	NP_058987.2	98.9	273	56.88	gamma-aminobutyric acid receptor subunit rho-1 precursor	13.2
TR92173|c7_g1_i6	NP_058988.1	96.81	408	87.74	gamma-aminobutyric acid receptor subunit rho-2 precursor	4.2
TR93490|c3_g2_i13	NP_068627.1	99	299	100	cone-rod homeobox protein	13.7
TR99284|c0_g4_i1	NP_114190.1	99.62	261	100	calbindin	3.5

Trinity transcripts were queried using BlastX against *R*. *norvegicus* RefSeq proteins. Results reported for selected retinal cell specific markers.

**Fig 4:**
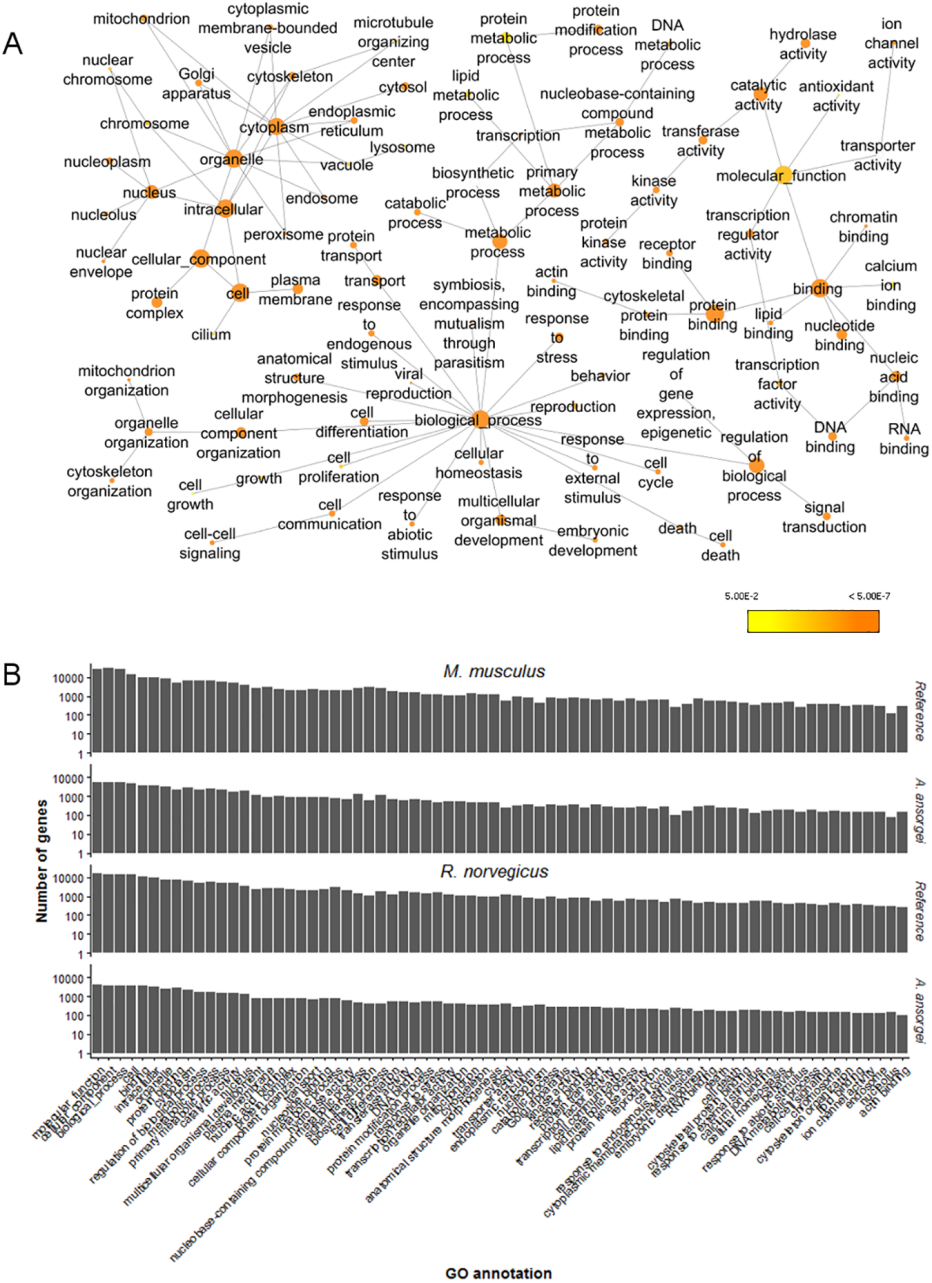
Enrichment analysis of Gene Ontology annotations. A) Enrichment of GOSlim annotations in the molecular function, cellular component, and molecular process subgroups. Nodes are enriched GO annotations, and their sizes are proportional to the number of genes with which they are associated. Color scale indicates Benjamin-Hochberg False Discovery Rate (FDR) corrected p-value from hypergeometric test for enrichment. B) Number of genes corresponding to GOSlim annotations with greater than 2% coverage for *A*. *ansorgei* as compared to both *M*. *musculus* and *R*. *norvegicus* references.

BiNGO was used to identify the GO annotations within the GOSlim subgroup enriched among the Trinity transcripts with Swiss-Prot BlastX homology expressed at greater than 1 TPM using *R*. *norvegicus* as the reference (Fig 4A). The number of genes corresponding to GOSlim annotations with greater than 2% coverage was assessed. Although the absolute number of genes is lower for *A*. *ansorgei* than for either the *M*. *musculus* reference or the *R*. *norvegicus* reference, the relative rank order for the GO annotation coverage is similar between *A*. *ansorgei* and both references (Fig 4B). The top GO annotations are broadly distributed amongst the molecular function, cellular component, and biological process subgroupings.

## Discussion

We performed RNA-Seq on adult retinas from the Nile grass rat *A*. *ansorgei* and used Trinity to de novo assemble and functionally annotate the first high quality draft transcriptome of this species. The assembly had an N50 length of 1,457 nt and included 400,584 transcripts, of which 46,038 were greater than 1kb in length and demonstrated Swiss-Prot BlastX homology. As expected, phylogenetic analysis placed *A*. *ansorgei* in closer evolutionary proximity to *M*. *musculus* and *R*. *norvegicus* than to other common model organisms, including *C*. *porcellus* (guinea pig). Trinity transcripts conferred full or near full length coverage of 14,397 *M*. *musculus* and 13,095 *R*. *norvegicus* RefSeq proteins, including highly conserved housekeeping genes and retinal cell specific markers. Pairwise comparison of retinal gene expression for *A*. *ansorgei* and *A*. *niloticus* showed that global transcriptome profiles were moderately correlated between these two Arvicanthis genus members.

Taken together, the findings suggest that our draft transcriptome is high-quality with respect to diversity, contiguity, and coverage. Global scale species specific sequence information was previously non-existent for *A*. *ansorgei*, limiting the capacity for molecular based studies. The *A*. *ansorgei* retinal transcriptome has now been made publicly available. Our hope is that it may serve the broader research community and provide a foundation for the use of *A*. *ansorgei* as a model organism for future cellular and molecular investigations related to cone biology and retinal degeneration and for comparison to other common model organisms, including *M*. *musculus* and *R*. *norvegicus*.

## Supporting information

S1 Fig Bioanalyzer RNA 6000 Nano for RNA used for library preparation. A) S1 RIN 9.8; B) S2 RIN 9.7.
(TIF)

S2 Fig High sensitivity DNA Bioanalyzer for sequenced Nextera RNA-Seq libraries. A) S1; B) S2.
(TIF)

S3 Fig FastQC per base sequence quality. A) S1 read 1; B) S1 read 2; C) S2 read 1; D) S2 read 2.
(TIF)

S4 Fig Pairwise gene expression comparison between *A*. *ansorgei* and *A*. *niloticus*. 8,866 orthologous genes expressed at a level greater than 1 TPM (*A*. *ansorgei*) or 1 FPKM (*A*. *niloticus*).
(TIFF)

S1 File Assembled sequences for *A*. *ansorgei* retinal transcriptome. (RAR)

S2 File Annotations and expression levels for transcripts from *A*. *ansorgei* de novo assembly. (RAR)

## References

[1] Carter-Dawson LD, LaVail MM. Rods and Cones in the Mouse Retina I. Structural Analysis Using Light and Electron Microscopy. J Comp Neurol. 1979;188: 245–262. doi: 10.1002/cne.901880204 500858 10.1002/cne.901880204

[2] Szel A, Rohlich P. Two Cone Types of Rat Retina Detected by Anti-visual Pigment Antibodies. Exp Eye Res. 1992;55: 47–52. 1397129 10.1016/0014-4835(92)90090-f

[3] Caldelas I, Poirel VJ, Sicard B, Pévet P, Challet E. Circadian profile and photic regulation of clock genes in the suprachiasmatic nucleus of a diurnal mammal Arvicanthis ansorgei. Neuroscience. 2003;116: 583–591. doi: 10.1016/S0306-4522(02)00654-1 12559113 10.1016/s0306-4522(02)00654-1

[4] Van Hooser SD, Nelson SB. The squirrel as a rodent model of the human visual system. Vis Neurosci. 2006;23: 765–78. doi: 10.1017/S0952523806230098 17020632 10.1017/S0952523806230098

[5] Merriman DK, Lahvis G, Jooss M, Gesicki JA, Schill K. Current practices in a captive breeding colony of 13-lined ground squirrels (Ictidomys tridecemlineatus). Lab Anim. Nature Publishing Group; 2012;41: 315–325. doi: 10.1038/laban.150 23079915 10.1038/laban.150

[6] Bobu C, Craft CM, Masson-Pevet M, Hicks D. Photoreceptor organization and rhythmic phagocytosis in the nile rat Arvicanthis ansorgei: A novel diurnal rodent model for the study of cone pathophysiology. Investig Ophthalmol Vis Sci. 2006;47: 3109–3118. doi: 10.1167/iovs.05-1397 16799057 10.1167/iovs.05-1397 PMC2933834

[7] Boudard DL, Tanimoto N, Huber G, Beck SC, Seeliger MW, Hicks D. Cone loss is delayed relative to rod loss during induced retinal degeneration in the diurnal cone-rich rodent Arvicanthis ansorgei. Neuroscience. Elsevier Inc.; 2010;169: 1815–1830. doi: 10.1016/j.neuroscience.2010.06.037 20600653 10.1016/j.neuroscience.2010.06.037

[8] Castiglia R, Bekele A, Makundi R, Oguge N, Corti M. Chromosomal diversity in the genus Arvicanthis (Rodentia, Muridae) from East Africa: A taxonomic and phylogenetic evaluation. J Zool Syst Evol Res. 2006;44: 223–225. doi: 10.1111/j.1439-0469.2006.00356.x

[9] Mustafi D, Kevany BM, Bai X, Golczak M, Adams MD, Wynshaw-Boris A, et al Transcriptome analysis reveals rod/cone photoreceptor specific signatures across mammalian retinas. Hum Mol Genet. 2016;25: 4376–4388. doi: 10.1093/hmg/ddw268 28172828 10.1093/hmg/ddw268 PMC6078599

[10] Dobigny G, Tatard C, Gauthier P, Ba K, Duplantier J, Granjon L, et al Mitochondrial and Nuclear Genes-Based Phylogeography of Arvicanthis niloticus (Murinae) and Sub-Saharan Open Habitats Pleistocene History. PLoS One. 2013;8: e77815 doi: 10.1371/journal.pone.0077815 24223730 10.1371/journal.pone.0077815 PMC3815218

[11] Ducroz J-F, Volobouev V, Granjon L. A Molecular Perspective on the Systematics and Evolution of the Genus Arvicanthis (Rodentia, Muridae): Inferences from Complete Cytochrome b Gene Sequences. Mol Phylogenet Evol. 1998;10: 104–117. doi: 10.1006/mpev.1997.0477 9751921 10.1006/mpev.1997.0477

[12] Farkas MH, Grant GR, White J a, Sousa ME, Consugar MB, Pierce E a. Transcriptome analyses of the human retina identify unprecedented transcript diversity and 3.5 Mb of novel transcribed sequence via significant alternative splicing and novel genes. BMC Genomics. 2013;14: 486 doi: 10.1186/1471-2164-14-486 23865674 10.1186/1471-2164-14-486 PMC3924432

[13] Bolger AM, Lohse M, Usadel B. Trimmomatic: a flexible trimmer for Illumina sequence data. Bioinformatics. 2014;30: 2114 doi: 10.1093/bioinformatics/btu170 24695404 10.1093/bioinformatics/btu170 PMC4103590

[14] seq crumbs. In: Bioinformatics at COMAV [Internet]. [cited 3 May 2016]. Available: https://bioinf.comav.upv.es/seq_crumbs/

[15] Grabherr MG, Haas BJ, Yassour M, Levin JZ, Thompson D a, Amit I, et al Full-length transcriptome assembly from RNA-Seq data without a reference genome. Nat Biotechnol. 2011;29: 644–652. doi: 10.1038/nbt.1883 21572440 10.1038/nbt.1883 PMC3571712

[16] Li B, Dewey CN. RSEM: accurate transcript quantification from RNA-Seq data with or without a reference genome. BMC Bioinformatics. 2011;12: 323 doi: 10.1186/1471-2105-12-323 21816040 10.1186/1471-2105-12-323 PMC3163565

[17] Camacho C, Coulouris G, Avagyan V, Ma N, Papadopoulos J, Bealer K, et al BLAST+: architecture and applications. BMC Bioinformatics. 2009;10: 421 doi: 10.1186/1471-2105-10-421 20003500 10.1186/1471-2105-10-421 PMC2803857

[18] The UniProt Consortium. UniProt: a hub for protein information. Nucleic Acids Res. 2014;43: D204–12. doi: 10.1093/nar/gku989 25348405 10.1093/nar/gku989 PMC4384041

[19] Eddy SR. Accelerated profile HMM searches. PLoS Comput Biol. 2011;7 doi: 10.1371/journal.pcbi.1002195 22039361 10.1371/journal.pcbi.1002195 PMC3197634

[20] Finn RD, Coggill P, Eberhardt RY, Eddy SR, Mistry J, Mitchell AL, et al The Pfam protein families database: towards a more sustainable future. Nucleic Acids Res. 2015;44: D279–D285. doi: 10.1093/nar/gkv1344 26673716 10.1093/nar/gkv1344 PMC4702930

[21] Petersen TN, Brunak S, von Heijne G, Nielsen H. SignalP 4.0: discriminating signal peptides from transmembrane regions. Nat Methods. Nature Publishing Group; 2011;8: 785–786. doi: 10.1038/nmeth.1701 21959131 10.1038/nmeth.1701

[22] Krogh A, Larsson B, von Heijne G, Sonnhammer ELL. Predicting transmembrane protein topology with a hidden Markov model: Application to complete genomes. J Mol Biol. 2001;305: 567–580. doi: 10.1006/jmbi.2000.4315 11152613 10.1006/jmbi.2000.4315

[23] Lagesen K, Hallin P, Rødland EA, Stærfeldt HH, Rognes T, Ussery DW. RNAmmer: Consistent and rapid annotation of ribosomal RNA genes. Nucleic Acids Res. 2007;35: 3100–3108. doi: 10.1093/nar/gkm160 17452365 10.1093/nar/gkm160 PMC1888812

[24] Suzek BE, Huang H, McGarvey P, Mazumder R, Wu CH. UniRef: Comprehensive and non-redundant UniProt reference clusters. Bioinformatics. 2007;23: 1282–1288. doi: 10.1093/bioinformatics/btm098 17379688 10.1093/bioinformatics/btm098

[25] Blake JA, Christie KR, Dolan ME, Drabkin HJ, Hill DP, Ni L, et al Gene ontology consortium: Going forward. Nucleic Acids Res. 2015;43: D1049–D1056. doi: 10.1093/nar/gku1179 25428369 10.1093/nar/gku1179 PMC4383973

[26] Powell S, Forslund K, Szklarczyk D, Trachana K, Roth A, Huerta-Cepas J, et al EggNOG v4.0: Nested orthology inference across 3686 organisms. Nucleic Acids Res. 2014;42: 231–239. doi: 10.1093/nar/gkt1253 24297252 10.1093/nar/gkt1253 PMC3964997

[27] Subramanian A, Tamayo P, Mootha VK, Mukherjee S, Ebert BL, Gillette M a, et al Gene set enrichment analysis: a knowledge-based approach for interpreting genome-wide expression profiles. Proc Natl Acad Sci U S A. 2005;102: 15545–50. doi: 10.1073/pnas.0506580102 16199517 10.1073/pnas.0506580102 PMC1239896

[28] Shannon P, Markiel A, Ozier O, Baliga NS, Wang JT, Ramage D, et al Cytoscape: a software environment for integrated models of biomolecular interaction networks. Genome Res. 2013;13: 2498–2504. doi: 10.1101/gr.1239303.metabolite 10.1101/gr.1239303 PMC403769 14597658

[29] Edgar RC. MUSCLE: Multiple sequence alignment with high accuracy and high throughput. Nucleic Acids Res. 2004;32: 1792–1797. doi: 10.1093/nar/gkh340 15034147 10.1093/nar/gkh340 PMC390337

[30] Saitou N NM. The Neighbor-joining Method: A New Method for Reconstructing Phylogenetic Trees’. Mol Biol Evol. 1987;4: 406–425. citeulike-article-id:93683 3447015 10.1093/oxfordjournals.molbev.a040454

[31] Tamura K, Nei M, Kumar S. Prospects for inferring very large phylogenies by using the neighbor-joining method. Proc Natl Acad Sci U S A. 2004;101: 11030–5. doi: 10.1073/pnas.0404206101 15258291 10.1073/pnas.0404206101 PMC491989

[32] Tamura K, Stecher G, Peterson D, Filipski A, Kumar S. MEGA6: Molecular evolutionary genetics analysis version 6.0. Mol Biol Evol. 2013;30: 2725–2729. doi: 10.1093/molbev/mst197 24132122 10.1093/molbev/mst197 PMC3840312

