# Supplementary figures and images for: De novo assembly and annotation of the retinal transcriptome for the Nile grass rat (Arvicanthis ansorgei)
Source: PLoS One. 2017 Jul 31;12(7):e0179061. doi: 10.1371/journal.pone.0179061 (PMC5536302; doi:10.1371/journal.pone.0179061)

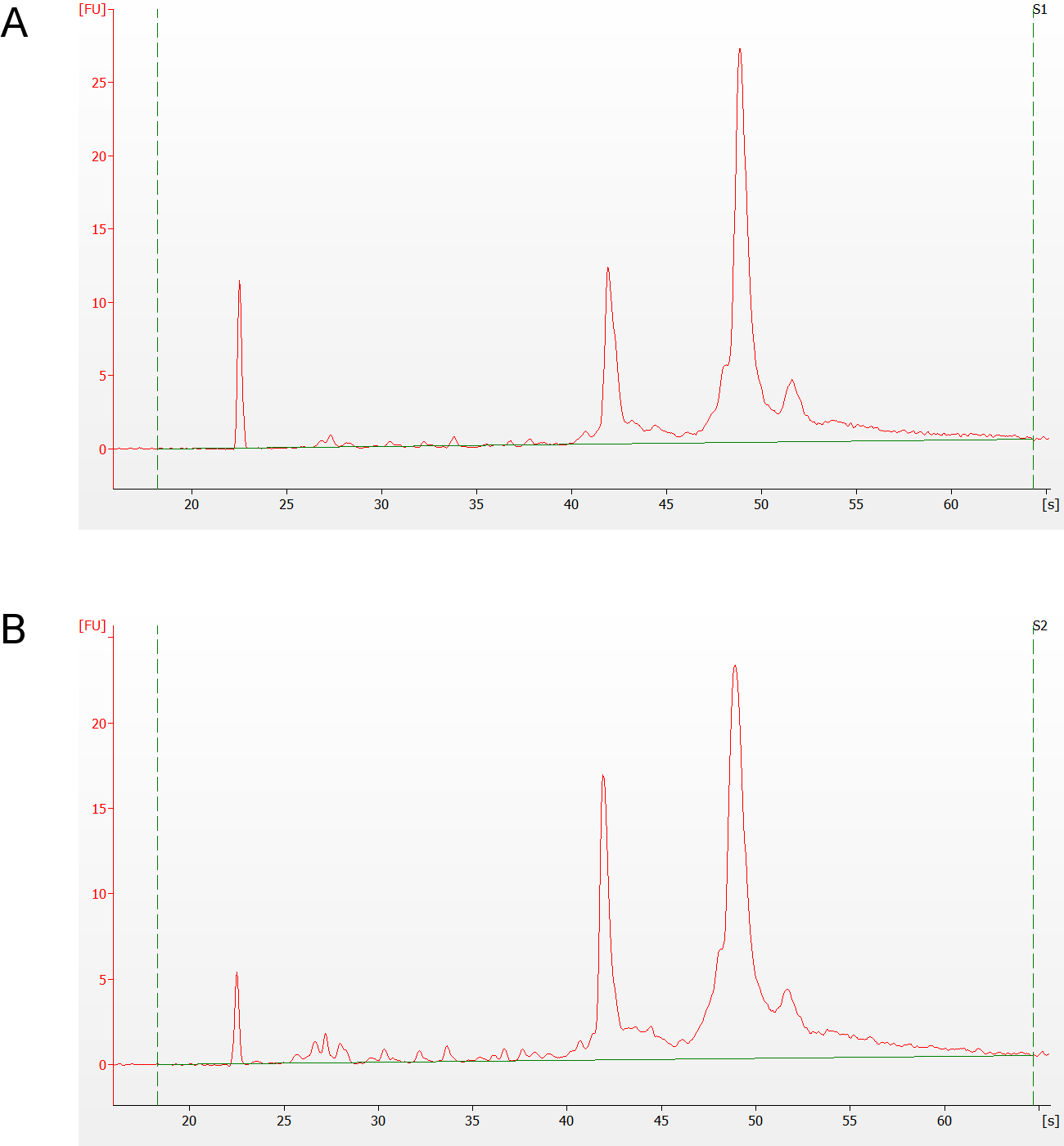

Supplement: S1 Fig — A) S1 RIN 9.8; B) S2 RIN 9.7. (TIF) [file pone.0179061.s001.tif]

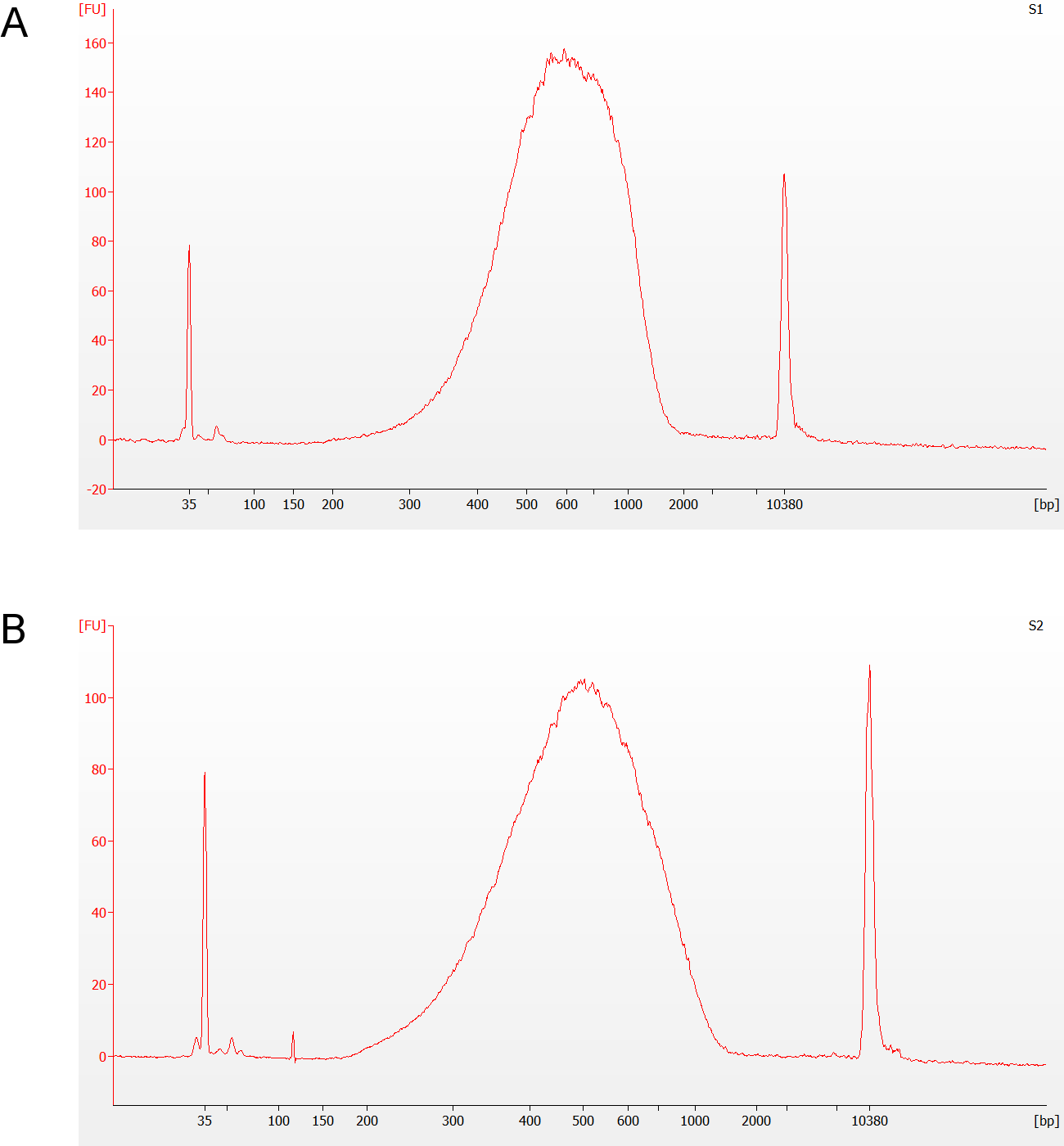

Supplement: S2 Fig — A) S1; B) S2. (TIF) [file pone.0179061.s002.tif]

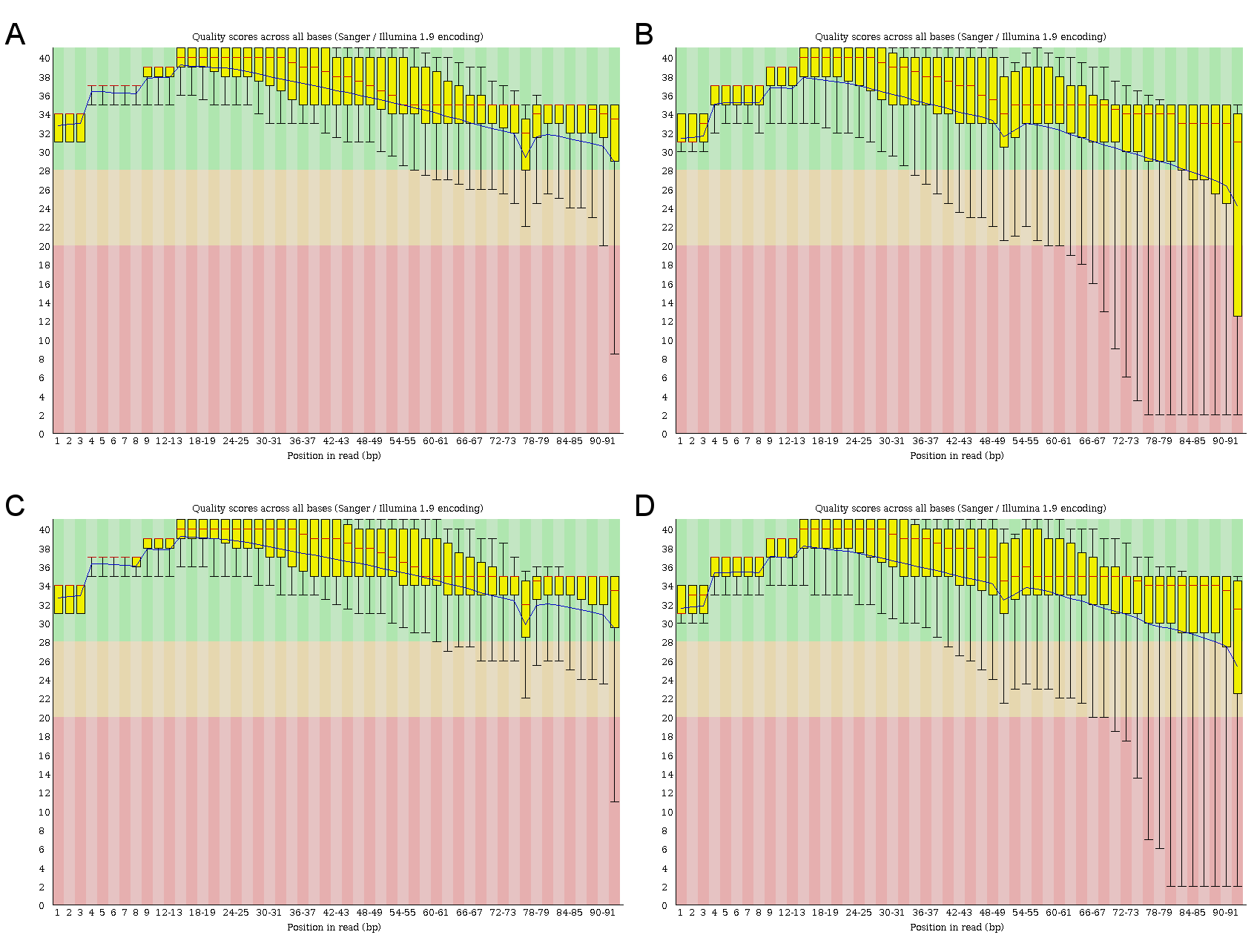

Supplement: S3 Fig — A) S1 read 1; B) S1 read 2; C) S2 read 1; D) S2 read 2. (TIF) [file pone.0179061.s003.tif]

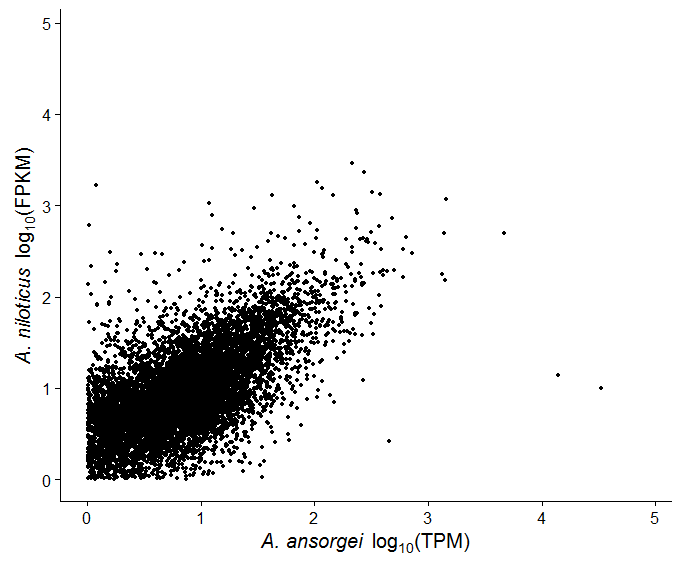

Supplement: S4 Fig — 8,866 orthologous genes expressed at a level greater than 1 TPM (A. ansorgei) or 1 FPKM (A. niloticus). (TIFF) [file pone.0179061.s004.tiff]
